# Non-Invasive Solutions to Identify Distinctions Between Healthy and Mild Cognitive Impairments Participants

**DOI:** 10.1109/JTEHM.2022.3175361

**Published:** 2022-05-16

**Authors:** Eaman A. Alharbi, Janelle M. Jones, Akram Alomainy

**Affiliations:** School of Electronic Engineering and Computer ScienceQueen Mary University of London 4617 London E1 4NS U.K.; School of Biological and Behavioural SciencesQueen Mary University of London 4617 London E1 4NS U.K.

**Keywords:** Mild cognitive impairment, dementia, HRV, non-invasive techniques, sensors

## Abstract

Mild cognitive impairment (MCI) is a condition characterized by impairment in a single cognitive domain or mild deficit in several cognitive domains. MCI patients are at increased risk of progression to dementia with almost 50% of MCI patients developing dementia within five years. Early detection can play an important role in early intervention, prevention, and appropriate treatments. In this study, we examined heart rate variability (HRV) as a novel physiological biomarker for identifying individuals at higher risk of MCI. We investigated if measuring HRV using non-invasive sensors might offer reliable, non-invasive techniques to distinguish MCI patients from healthy controls. Twenty-one MCI patients were recruited to examine this possibility. HRV was assessed using CorSense wearable device. HRV indices were analyzed and compared in rest between MCI and healthy controls. The significance of difference of numerical data between two groups was assessed using parametric unpaired t-test or non-parametric Wilcoxon rank sum test based on the fulfilment of unpaired t-test assumptions. Multiple linear regression models were performed to assess the association between individual HRV parameter with the cognitive status adjusting for gender and age. Time-domain parameters i.e., the standard deviation of NN intervals (SDNN), and the root mean square of successive differences between normal heartbeats (RMSSD) were significantly lower in MCI patients compared with healthy controls. Prediction accuracy for the logistic regression using 10-fold cross-validation was 76.5%, Specificity was 0.8571, while sensitivity was 0.8095. Our study demonstrated that healthy participants have higher HRV indices compared to older adults with MCI using non-invasive biosensors technologies. Our results are of clinical importance in terms of showing the possibility that MCI of older people can be predicted using only HRV PPG-based data.

## I. Introduction

Mild Cognitive Impairment (MCI) is a stage of cognitive decline that occurs between the expected cognitive decline associated with healthy aging and the decline seen in dementia. Individuals with MCI experience memory loss or other cognitive domain losses such as language deficits whilst they still maintain their ability to independently perform in daily living activities. MCI is recognized as an important public health problem as a dementia risk [1]. The rate at which those diagnosed with MCI progress to dementia is 3 to 5 times higher than for those with normal cognition [2], [3] with 12% rate of annual progression in the general population and up to 20% in populations that are at higher risk [1], [4]. Dementia is still diagnosed primarily on clinical signs. Biomarkers, on the other hand, are being increasingly recognised as having a significant role to play [5]. Biomarkers and digital biomarkers for dementia can be a promising approach for early-stage pathological diagnosis of dementia since they help objectively assess pathological sequences and disease progression.

Heart rate variability (HRV) is the measure of variations in the time between each heartbeat. This variation is controlled by the autonomic nervous system (ANS). HRV is considered as a valid and reliable diagnostic tool of autonomic regulation, including activation of the parasympathetic and sympathetic nervous systems [6]. Both systems are important for modulating many vital functions, including respiration and cardiac contractility. Low HRV is associated with emotional dysregulation, worse cognitive performance and is a well-established biomarker of cardiovascular disease [7]. HRV analysis provides an accurate, real-time, and non-invasive way to assess autonomic functioning and as such has been widely used in clinical research.

Conventionally HRV is obtained using one of the two widely used methods to measure the cardiac cycle which is electrocardiography (ECG), and photoplethysmography (PPG). For years, ECG has been used as dominant cardiac monitoring and to detect any abnormalities. However, until now, ECG haven’t been improved to the point where they can offer the user with flexibility, portability, and convenience. Whilst. PPG is a non-invasive tool uses light-based technology to measure the volumetric variations of blood circulation. PPG has proven to be a viable alternative to traditional HR monitoring when measured at rest [8].The usage of PPG sensors has increased due to non-invasiveness, ease of use, cost effectiveness, and it can be easily integrated into wearable wrist and finger-worn devices [9], [10]. PPG sensors are typically attached to the fingers because of the large amplitude that may be achieved as compared to other places. However, adopting PPG-based monitoring approaches can have some limitations such as inaccuracy in tracking PPG signals during everyday routine activities and light physical exercises. In fact, many studies have demonstrated that PPG – based devices are accurate and reliable for HRV during resting conditions [11], [12]. Specifically, PPG signal or pulse rate variability (PRV) acquired from the finger were the most similar to heart rate variability [13].

An association between HRV and cognitive function has been demonstrated in large cohorts of older patients as well as in smaller samples of subjects affected by dementia [14]. The first study to establish a correlation between HRV and cognitive functions emphasized changes in HRV based on the type or complexity of the cognitive task [15], [16]. On the basis of this work, several theories were developed to explain the link between HRV and cognitive functioning including the Neurovisceral Integration Model [17], which suggests that the brain areas engaged in cognitive and emotional functions are also involved in the regulation of autonomic function.

Within this perspective, HRV can play a significant role as a non-invasive and real-time accurate way to assess autonomic regulation. Several studies have demonstrated the predictive value as well as the clinical application of the HRV as a biomarker. Reduced HRV is considered to be a predictor for general mortality [18] and cognitive performance [19]. Furthermore, higher HRV was found to be associated with better cognitive performance, and a lower HRV has been associated with cognitive impairment [20]. Thus, measurement of HRV may add important information to an assessment of older adults’ cognitive function.

The present study will use off-the-shelf HRV monitor devices to assess whether real-time measures of HRV can be used as an early indicator of cognitive decline in individuals with MCI who still have intact cognitive abilities relative to healthy controls. We hypothesise that HRV indices will be lower among individuals with MCI relative to healthy controls. If these patterns emerge, it may be possible to identify biomarkers that could help in the detection of the disease in the preclinical stage which could facilitate an earlier intervention and early access to medical treatments to slow down the progression of the disease [21].

## II. Related Work

Our pilot work [22], demonstrated the feasibility of using wearables to assess relationships between autonomic and cognitive functioning. Here we recruited 10 (five males and five females) healthy young participants (M age = 28.6 years, SD = 2.50). We wanted to prove that wearable and sensors devices have the ability to identify and record physical data and do so reliably. A statistically significant difference was observed in the frequency-domain HRV measure HFnu measured prior to the Stroop test and that measured during it. The reduced HFnu during the test indicates the decreased parasympathetic activity during stress. Moreover, the LF/HF ratio significantly increased throughout the test implying an increase in the relative predominance of sympathetic nervous system activity during the test.

## III. Methods and Analysis

### A. Participants

A statistical power analysis was performed for sample size estimation, using the G*Power computer software [23]. A total sample of 18 people would be needed to detect large effects (d =.8 with 85% power for an independent-groups comparison with alpha at.05. Participants were 21 individuals with MCI and 21 healthy controls (MAge = 72.95 years, SD = 5.86, Range = 62–87; 14 male 28 female) recruited from Join Dementia Research (JDR). JDR is a service managed by the National Institute for Health Research in partnership with Alzheimer’s Society, Alzheimer’s Research UK, and Alzheimer Scotland. It allows people to register their interest in taking part in dementia research. JDR has demonstrated benefits in terms of increased research recruitment efficiency, access to research for the public and for researchers, public engagement in dementia and research participation [24]. Since its inception in early 2015, UK JDR has signed up over 50,000 volunteers and enrolled over 33,000 individuals into over 300 dementia studies. Over 1750 researchers are registered users, spanning over 296 National Health Service, university, and commercial research sites [25]. Participants were eligible for the present research if they were aged 60-90 and had a diagnosis of MCI (for the MCI group). Exclusion criteria included a diagnosis of a neurological condition or a Mini-Mental State Examination Score (MMSE) < 24. Participants with current alcohol or substance misuse, a history of cardiovascular conditions including stroke, ischemic attack, and other types of irregular rhythm disturbances, including atrial fibrillation and other arrhythmias were also excluded. Ethical approval was obtained from Queen Mary Ethics of Research Committee (QMERC20.210). All participants provided written informed consent prior to study completion.

### B. Data Collection

Data collection including questionnaire and HRV was carried out in a quiet room, between 8:30 a.m. and 12:00 p.m. since HRV can be affected by changes in circadian rhythm, hormonal shifts, and acute stressors throughout the day. Participants were instructed to eat a light breakfast and were asked to abstain from smoking and drinking any caffeine-containing beverages including tea and coffee for 2 hours prior to the assessments, and to refrain from drinking alcohol in the 12 hours prior to assessments. HRV was assessed for 6 minutes at rest, comprising a 1-minute stabilization period followed by 5 minutes of actual readings, in line with the recommendations of the Task Force of The European Society of Cardiology and The North American Society of Pacing and Electrophysiology [26]. We used the CorSense finger-worn device which has been proven to be a very accurate consumer-grade HRV monitor. The CorSense has been internally validated with accuracy equivalent to a 5-lead ECG/EKG, the gold standard for HRV detection, with less than 3% variation across multiple subjects with differing skin tones. CorSense measures heart rate variability through pulse detection using a gold-standard 500 hertz multiwave sensor array that conveniently and comfortably slips over participant’s finger. We have used Elite HRV Smartphone Application app to read the data and Kubios HRV 3.3.1 (Kubios Oy, Kuopio, Finland) software to analyse the data [27], [28].

### C. Statistical Analysis

Numerical data were expressed as mean ± standard deviation (SD) and median with interquartile range (IQR). Categorical data were expressed as frequency and percentages. The significance of difference of numerical data between two groups was assessed using parametric unpaired t-test or non-parametric Wilcoxon rank sum test based on the fulfilment of unpaired t-test assumptions (normality and equal variances). The significance of differences in categorical variables between groups was assessed using Chi-square test or Fisher’s exact test.

Multiple linear regression models were performed to assess the association between individual HRV parameter as an outcome and cognitive status as the independent variable, adjusting for age and gender. Age was dichotomized using the median value before being included in the models. Moreover, we ran a logistic regression analysis using measures significantly different between MCI patients and healthy controls to predict health status of each participant. This model included age, gender, mean RR, ln(SDNN), ln(RMSSD), and ln(HF) as predictor variables, and grouping variable (MCI vs. healthy control) as an outcome variable. We ran a 10-fold cross-validation to compute model prediction accuracy. Individuals were initially classified into MCI/healthy groups based on a threshold of 0.5, which means that all individuals with predicted probability of MCI over 0.5 were classified as MCI patients, and individuals with predicted probability of MCI below 0.5 were classified as healthy controls. Sensitivity and specificity indices were calculated for this threshold value and receiver operating characteristic (ROC) curve was created for each value of the threshold All statistical analysis was conducted in R software (version 4.1.2; R Foundation for Statistical Computing, Vienna, Austria) [29].

### D. Result

The mean age of MCI subjects was significantly greater than that of healthy subjects (74.9 ± 5.43 vs. 71 ± 5.75 years). As depicted in Table 1, the cognitive status was not associated with gender, smoking status, physical activity, or educational level (p = 0.513, 0.488, 0.739, and 0.564, respectively). Regarding the time domain parameters, the mean RR time was significantly different between healthy and MCI subjects (920 ± 90.2 vs. 898 ± 195.4 ms, respectively). For the time-domain indices, both SDNN and RMSSD were significantly lower in MCI subjects compared with healthy subjects (p = 0.014 and 0.004, respectively). “Of the frequency-domain parameters, only HF showed a statistically significant difference between the two groups (p = 0.055). Differences in other indices, including VLF, LF, and LF/HF ratio, between healthy and MCI subjects were not statistically significant. Prediction accuracy for the logistic regression using 10-fold cross-validation was 76.5%. Specificity of the full model was 0.8571, while sensitivity was 0.8095. Highest accuracy of the model was achieved at a threshold of 0.5. ROC curve showing classification performance at values of all thresholds is presented in Fig. 6.

**TABLE 1:** Demographic Data and Summary Statistics of HRV Parameters in Study Subjects

	Healthy n = 21	MCI n = 21	P-value
Age, years Mean (SD)	71 (5.75)	74.9 (5.43)	0.029^a^
Median (IQR)	71 (66-74)	76 (72-77)	
Gender, n (%) Female	15 (71.4%)	13(61.9%)	0.513^b^
Male	6 (28.6%)	8 (38.1%)	
Smoking status, n (%) Non-smoker	21 (100%)	19 (90.5%)	0.488^c^
Smoker	0 (0%)	2 (9.5%)	
Physical activity, n (%) Not active	6 (28.6%)	7 (33.3%)	0.739^b^
Active	15 (71.4%)	14 (66.7%)	
Education level, n (%) School	4 (19%)	6 (28.6%)	0.564^b^
Undergraduate	8 (38.1%)	5 (23.8%)	
postgraduate Mean RR, ms	9 (42.9%)	10 (47.6%)	
Mean (SD)	920 (90.2)	898 (195.4)	0.639^d^
Median (IQR)	933 (886-978)	873 (760-1003)	
SDNN, ms Mean (SD)	37.6 (18.8)	26.5 (15.8)	0.014^e^
Median (IQR)	31.6 (24.2-44)	22.3 (15.8-34.5)	
RMSSD, ms Mean (SD)	46.1 (23.4)	30.5 (25.7)	0.004^e^
Median (IQR)	44.4 (28.3-59)	19.1 (15.2-35.4)	
VLF, ms2 Mean (SD)	40.1 (46.4)	56.2 (68.7)	0.485^e^
Median (IQR)	22.5 (12.7-54.9)	28.8 (16.4-78.6)	
LF, ms2 Mean (SD)	477 (594)	289 (242)	0.672^e^
Median (IQR)	187.2 (106.7-622.6)	213.5 (121.4-419.7)	
HF, ms2 Mean (SD)	450 (461)	310 (544)	0.055^e^
Median (IQR)	310.7 (117-741)	151.4 (64.4-264.3)	
LF/HF Mean (SD)	1.3 (1.06)	1.91 (1.66)	0.229^e^
Median (IQR)	1.03 (0.53-1.64)	1.6 (0.77-2.54)	

^a^ Unpaired t-test,

^b^ Chi-square test,

^c^ Fisher’s Exact test,

^d^ Welch Unpaired t-test,

^e^ Wilcoxon rank sum test.

IQR: interquartile rang,

SD: standard deviation,

ms: milliseconds

**FIGURE 1.**
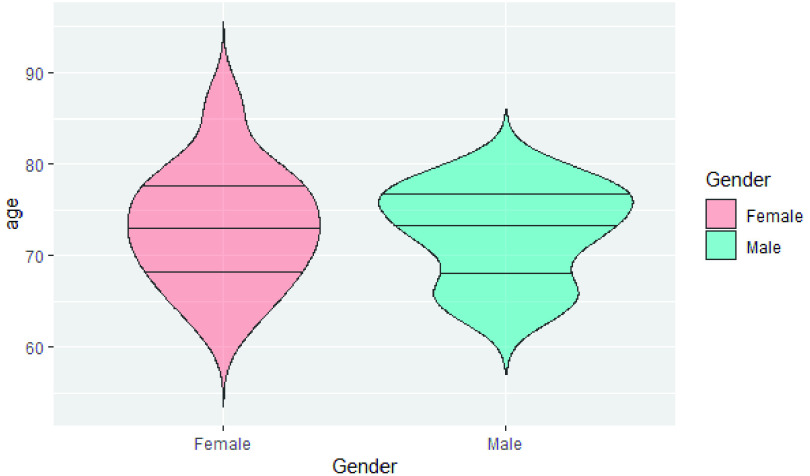
Age and gender distribution of the participants.

**FIGURE 2.**
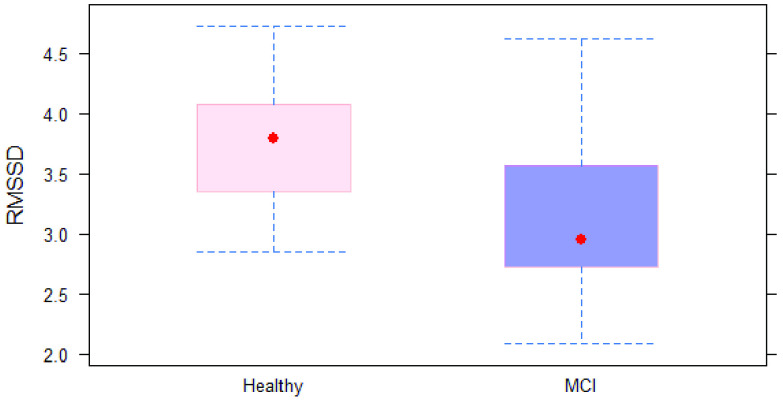
RMSSD in MCI and controls group.

**FIGURE 3.**
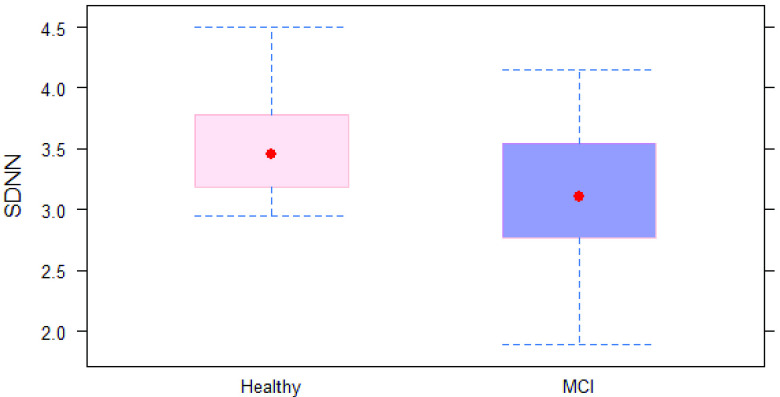
SDNN in MCI and controls group.

**FIGURE 4.**
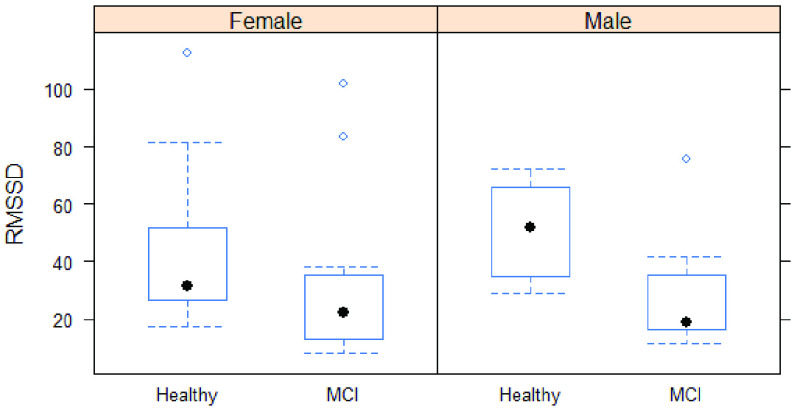
RMSSD when controlling for age and gender.

**FIGURE 5.**
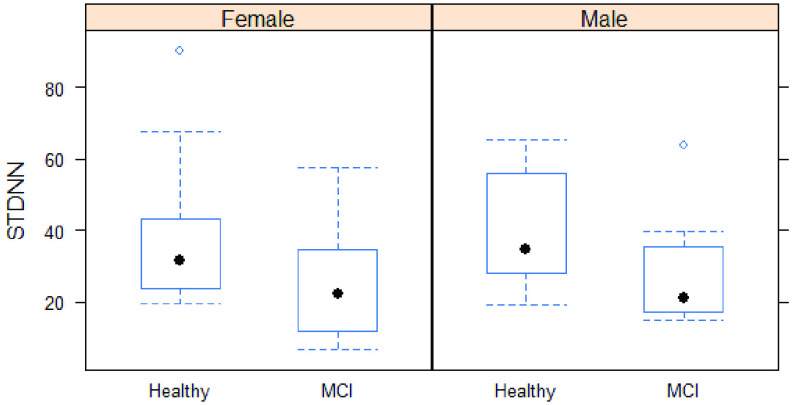
SDNN when controlling for age and gender.

**FIGURE 6.**
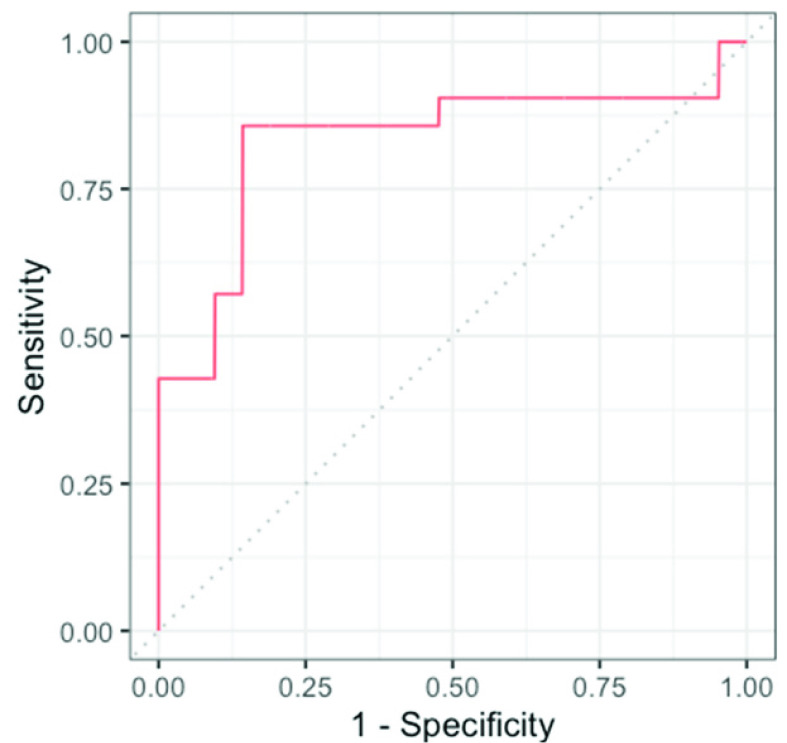
ROC curve for every value of classification threshold.

Different linear models were built to test the association between HRV parameters as dependent variables and cognitive status, as the independent variable controlling for both age and gender. Cognitive status was significantly associated with ln(SSDN) and ln(RMSSD) but was not significantly associated with mean RR after adjusting for age and gender. The MCI subjects had approximately 35% and 43% reduction in SDNN and RMSSD, respectively, compared to healthy subjects controlling for age and gender. Out of the frequency domain parameters, cognitive status was only significantly associated with ln(HF) after adjusting for gender age and gender. The MCI subjects showed approximately 58% reduction in HF compared with healthy subjects (p = 0.012).

## IV. Discussion

Several studies have investigated different biomarkers in order to diagnose and assess neurodegenerative disease and MCI using biosensors. However, these biomarkers are not ideal solutions for healthcare systems, because they are expensive, time-consuming, and invasive [30]. Furthermore, several attempts have been made to develop biomarkers for the diagnosis of MCI [31], [32]. Yet, there is still considerable scope for improvement in terms of accessibility, reliability, and validity of these biomarkers. To our knowledge, only one study has investigated the feasibility of using biosensor device in patients at risk for dementia. The participants were divided into three groups: 24 healthy controls, 6 had subjective cognitive deterioration, and 3 were amyloid-positive (one with pre-clinical AD, one with pre-clinical Lewy-Body Dementia, and one with mild cognitive impairment) [13].

In this study, we explored differences between HRV in MCI group and in healthy controls, as measured using a PPG sensor. We investigated the feasibility of employing sensors to distinguish between MCI participants and healthy participants. Our primary hypothesis was supported as we observed significant differences between subjects with MCI and cognitively normal controls. Conventional time-domain and frequency-domain measures have been used for HRV analysis in this study. There was a significant difference in three HRV indices (RMSSD, SDNN and HF) between the two groups. Our findings show reduced HRV indices, suggesting lower parasympathetic activity is associated with MCI participants. This suggests that the autonomic dysfunction represented by HRV is detectable in baseline conditions using PPG sensors. Our findings demonstrate that real-time measures of HRV could be used as an early indicator of cognitive decline in individuals with MCI. These findings could be valuable to researchers and clinicians considering using HRV measurement for evaluating neurodegenerative disease in a large population.

Overall, the individual regression results and logistic regression analysis show that RMSSD, SDNN, and HF measures can be used to reliably distinguish MCI patients from healthy controls. Average accuracy of 76.5% is high and a classification threshold of 0.5 yields high sensitivity and specificity. Area under the ROC curve shows that the test has a very good diagnostic accuracy [33]. Previous studies have shown that MCI is related to a dysregulation and changes in HRV [34]–[36]. This is related to a dysfunction of the autonomic nervous system. Altered function of the autonomic nervous system is also related to worse cognitive performance in the absence of dementia [20]. This knowledge has the potential to contribute to the diagnosis of MCI and other cognitive deficits. However, it has not been applied this way before. Our study is the first one to show that using biosensors to measure HRV can be relatively reliably to distinguish cognitively normal healthy controls from MCI patients. Because HRV can be measured in a matter of minutes, the knowledge that we present here might be particularly useful and, in the future and provided that more studies on the topic are conducted, contribute to a battery of tools used to diagnose MCI.

This study also has limitations that are worth mentioning, the study included a sample of predominantly white older adults (more than 81% of the participants are white), so our findings may not apply to other populations. Furthermore, we had a majority of female participants, which may have prevented us from detecting differences in HRV due to gender. However, gender differences in HRV have been reported to disappear after the age of 50 years. [37]. Moreover, the HRV measured from the participants who were already diagnosed with MCI and it’s worth mentioning that HRV can be considered as a biomarker for already-diagnosed MCI and that does not necessarily imply that it’s a useful biomarker for as-yet-undiagnosed MCI. Further, it is known that the within-subject variability in short-term measured HRV (5-15min) could be very high [38]. In fact, the coefficient of variation for such measurements can vary between 1-100%. There are several factors that might influence intraindividual HRV reliability, such as stress, taking part in a pharmacological intervention, or belonging to a clinical population [39], [38]. On the other hand, short term HRV measurements have a number of advantages, as they can be conducted quickly and are relatively easy to analyze, but they can also be performed in a highly controlled environment. This could alleviate some of the concerns related to high within-subject variability. Other than that, strategies exist to improve HRV reliability, such as reminding individuals to avoid irregular respiration [40], or using specific measures that are less prone to individual variability in HRV, such as time-domain measurements, as opposed to frequency-domain measurements [41], or taking HRV measures at rest. Given that HRV is less reliable in clinical populations, using measures to improve such reliability in MCI patients is especially important and could improve the sensitivity, specificity, and accuracy of distinguishing MCI patients from healthy controls. Finally, since HRV reliability is specific to a measured population, further studies in patients with MCI need to be conducted that would aim specifically at investigating reliability of HRV measurements in this population.

## V. Conclusion and Future Work

Overall, our study demonstrated that healthy participants have higher HRV indices compared to older adults with MCI using sensors technologies. SDNN, RMSSD, and HF were significantly lower in MCI subjects compared with healthy subjects. Our results are of clinical importance in terms of showing the possibility that MCI of older people can be predicted using only HRV data. It was a control study and limited and therefore further studies would be needed but this is a very good indication that HRV PPG sensors technologies have potential as a non-invasive early marker to detect those at higher risk of having MCI. Future studies should extend these findings by including individuals with Alzheimer’s disease to investigate whether HRV could be a useful diagnostic screening tool at MCI stage of dementia by following up with the participants and identify MCI patients who underwent HRV testing at baseline, and who developed dementia. Moreover, more studies are needed to evaluate the predictive value of HRV in the progression of cognitive decline and how this links to the likelihood of dementia conversion.
